# Paper 4: a review of reporting and disseminating approaches for rapid reviews in health policy and systems research

**DOI:** 10.1186/s13643-022-01897-5

**Published:** 2022-07-30

**Authors:** Shannon E. Kelly, Jessie McGowan, Kim Barnhardt, Sharon E. Straus

**Affiliations:** 1 School of Epidemiology and Public Health, University of Ottawa, Suite 101, 600 Peter Morand Crescent, ON K1G 5Z3 Ottawa, Canada; 2 Cardiovascular Research Methods Centre, University of Ottawa Heart Institute, 40 Ruskin Street, Ottawa, ON K1Y 4W7 Canada; 3 Communications, CMAJ, 1410 Blair Towers, Suite 500, ON K1J 9B9 Ottawa, Canada; 4 Li Ka Shing Knowledge Institute of St. Michael’s - Unity Health Toronto, 38 Shuter St, ON M5B 1A6 Toronto, Canada

**Keywords:** Rapid review, Time factors, Evidence synthesis, Methods, Reporting, Dissemination, Health services, Health policy

## Abstract

**Background:**

Transparent reporting of rapid reviews enables appropriate use of research findings and dissemination strategies can strengthen uptake and impact for the targeted knowledge users, including policy-makers and health system managers. The aim of this literature review was to understand reporting and dissemination approaches for rapid reviews and provide an overview in the context of health policy and systems research.

**Methods:**

A literature review and descriptive summary of the reporting and disseminating approaches for rapid reviews was conducted, focusing on available guidance and methods, considerations for engagement with knowledge users, and optimizing dissemination. MEDLINE, PubMed, Google scholar, as well as relevant websites and reference lists were searched from January 2017 to March 2021 to identify the relevant literature with no language restrictions. Content was abstracted and charted.

**Results:**

The literature review found limited guidance specific to rapid reviews. Building on the barriers and facilitators to systematic review use, we provide practical recommendations on different approaches and methods for reporting and disseminating expedited knowledge synthesis considering the needs of health policy and systems knowledge users. Reporting should balance comprehensive accounting of the research process and findings with what is “good enough” or sufficient to meet the requirements of the knowledge users, while considering the time and resources available to conduct a review. Typical approaches may be used when planning the dissemination of rapid review findings; such as peer-reviewed publications or symposia and clear and ongoing engagement with knowledge users in crafting the messages is essential so they are appropriately tailored to the target audience. Consideration should be given to providing different products for different audiences. Dissemination measures and bibliometrics are also useful to gauge impact and reach.

**Conclusions:**

Limited guidance specific to the reporting and dissemination of rapid reviews is available. Although approaches to expedited synthesis for health policy and systems research vary, considerations for the reporting and dissemination of findings are pertinent to all.

**Supplementary Information:**

The online version contains supplementary material available at 10.1186/s13643-022-01897-5.

## Background

A systematic review uses explicit and systematic methods to identify, select, critically appraise, and extract and analyze data from relevant research [1]. However, this is not always possible, and the need for timely evidence syntheses often necessitates the use of methodological compromises. Rapid review is a type of knowledge synthesis with similar components to the systematic review process but uses simplified methods and an accelerated approach to produce information to meet this need for timeliness [2]. These changes in a rapid evidence product may differ according to the needs of the end-user, the producer, and other variables. For research to be valuable to knowledge users (i.e., individuals who may use review findings to make a decision), it must be reported clearly and transparently. Given the methodological tailoring of rapid reviews, which helps to expedite the review timeline, it is important that reporting reflect protocol-driven decisions, processes, and findings. This enables uptake and appropriate use of research findings across a variety of knowledge users [3].

Rapid reviews are often requested or commissioned directly by health system decision-makers, a feature, which strengthens their usability to influence local health systems by directly addressing and informing urgent policy issues or questions [4, 5]. Findings from rapid reviews are often contextualized to a particular health system setting in response to objectives specified by a knowledge-user, which increases usefulness for decision-making. Although approaches to rapid review for health policy and systems research may vary, the general considerations for reporting and disseminating evidence syntheses findings likely apply to all [5]. These considerations may also reflect the range of ways that individuals prefer to consume knowledge, from detailed reports to higher level briefs or plain language summaries.

Reporting is an important part of research synthesis. Guidance can be found from the Enhancing the QUAlity and Transparency Of health Research (EQUATOR) Network [6], who maintain a collection of up-to-date reporting tools and information to support the reporting guidelines to improve the quality of research publications. However, reporting is often done poorly or inadequately [7]. There is evidence that evidence syntheses fail to consider the needs of end-users or fail to provide enough detail to allow stakeholders to determine whether the findings are meaningful or trustworthy [7, 8]. Similarly, one of the most challenging tasks facing evidence producers is translating research findings into accepted practice, which requires tailored dissemination and adoption, and often considerable effort [9]. Dissemination involves communicating research results for a specific or targeted audience, with the goal of maximizing both uptake and impact [3]. Although strides have been made in enhancing researchers’ understanding of the importance of dissemination and effective dissemination strategies, there has been limited research on the impact of various strategies [10]. The aim of this literature review was to summarize reporting and dissemination approaches specific to rapid reviews and to provide an overview in the context of health policy and systems research.

## Methods

We conducted a broad literature review to describe current knowledge available to inform reporting and disseminating approaches for rapid reviews. Although this is not a systematic review, the general overview of our methods process supports good practice and transparency.

### Eligibility criteria

We defined a rapid review as a knowledge synthesis product that uses components from the systematic review process which are simplified or omitted to produce information in a short period of time [11–13]. We included peer-reviewed and agency-produced publications focused on or addressing reporting and disseminating approaches for rapid evidence products.

### Information sources and search strategy

We searched PubMed, MEDLINE, and Google Scholar (first 100 records) using keywords (e.g., rapid review, expedited synthesis, time factors) broadly representing the range of nomenclature associated with rapid evidence products [2, 14]. Any study design, including other narrative or descriptive articles, were considered. Other relevant literature was identified by searching the reference lists of key rapid review literature [2, 11, 12, 15–17]. Websites of known rapid review producers [15, 17] identified in these publications were also consulted. No language restrictions were applied. Searches were originally conducted in January 2017 and were updated in July 2019 and March 2021. All retrieved records were screened by a single reviewer (SK).

### Data elements and charting process

Salient content relevant to the reporting or dissemination of rapid reviews were captured and entered in Microsoft Excel by a single reviewer (SK) and discussed with the other reviewers.

### Data synthesis

We descriptively summarized results while focusing on health policy and systems research to capture available guidance and methods, and considerations for engagement with knowledge users.

## Results

Several peer-reviewed articles relevant to rapid review which were located and examined for content relevant to reporting and dissemination or application to health policy and systems research [4, 5, 8, 11–26]. In these records, we found limited guidance specific to rapid review reporting and dissemination; therefore, we used our existing knowledge and experience of known barriers and facilitators to systematic review use [27] to suggest key elements of format and content to consider when drafting a rapid review report [28–33]. Similarly, few articles directly addressed the dissemination of rapid evidence products. As such, we describe and discuss typical approaches for planning the dissemination of research using a rapid review lens [3, 10, 34–39].

### Goals of research reporting and dissemination

Clear, complete, and transparent reporting supports and encourages constructive and useful uptake of any research product [33]. For evidence syntheses, including rapid reviews, reporting enables and promotes understanding of research results and contributes to the appropriate use of research findings by a variety of knowledge users, including policy-makers and health system administrators [7]. Given the methodological tailoring of rapid reviews often employed by evidence producers to shorten the review timeline, it is important that reporting accounts for all protocol-driven decisions, processes, and findings, which may influence their interpretation [14].

Research results should be communicated and presented through a planned and tailored dissemination process [40]. Consideration should be given to the wider settings where the research will be received and to the target audiences, with the overall goal of supporting and maximizing both uptake and impact [18]. Dissemination activities and tools are customized by considering the significance of the findings, dissemination goals, target audiences, and anticipated impact or influence of the rapid review. In particular, an intersectionality lens should be used when developing the dissemination approach from engagement of the relevant audiences through to development of key messages and delivery strategies [41–44]. Intersectionality explores the social factors (e.g., age, education, gender identity) and their interaction with compounding power structures (e.g., education systems) and forms of discrimination (e.g., racism, ageism).

### Guidance and methods for reporting rapid reviews

#### Core principles of reporting knowledge syntheses

Inadequate or unclear reporting can potentially reduce the utility of a knowledge synthesis product if the knowledge users do not have enough information to evaluate the strengths and weaknesses of the synthesis process and/or the results [28]. Regardless of methods used for a rapid review, maintaining research integrity depends upon a few core principles to guide the processes of conducting the review and preparing its report [4]. Knowledge users are interested in both the findings of the review and its methods. Like other knowledge synthesis approaches, authors must limit reporting bias, by ensuring the protocol and any amendments are included in the final report [29, 30]. In general, authors of a rapid review should follow a few core principles (Table 1): *work from a protocol, use clear language*, *focus on reproducibility*, *and summarize strengths and weaknesses of the approach*, *take knowledge user needs into consideration.* If these basic principles are not adhered to, the knowledge user may lack adequate information to determine the reliability or validity of the review as a guide to decision-making. When methods are not described in acceptable detail, or when study findings are presented vaguely, selectively, and/or incompletely, the findings may be unusable [33]. This is wasteful for both the evidence producer and the knowledge user, and the health system, from a resource perspective [7]. Where an organization follows a common methodological approach for a suite of offered rapid evidence products, standardized methods may not always be fully reported in report; however, links to methodological documentation should be provided [20].

**Table 1:** Core principles for rapid review authors

Core principles [1, 2, 14, 18, 25, 32, 45, 46]
• Work from a protocol, and use it to guide the conduct and reporting of the review;
• Accurately and transparently document all steps, tailoring of methods, and judgements in the review process (such as: “Did the rapid review team make any methodological concessions to answer the research question[s] within available resources?”);
• Use clear language that will be understandable to knowledge users. Write at a level that someone without a university degree can understand, and avoid the use of jargon or technical terms, except where such terms are essential. Be aware of technical terminology or terms that may have a slightly different definition in the review setting than in everyday usage (e.g. blinding, control, practice);
• Provide enough detail in the account of the review methods so that a knowledgeable reader could reproduce the review;
• Summarize the methodological strengths and weaknesses using language designed to help non-experts interpret and judge the value of the review;
• Consider the needs of the knowledge user. Discuss their policy window, how findings will be reported early in the review process, whether a specific template is desired or required for reporting, or whether they have additional requirements beyond a traditional research findings report (e.g. a slide deck or policy brief). It may be helpful to provide the template to report the rapid review to knowledge users, and to ask if it meets their needs or if additional (or less) detail is needed. A rapid review report should be tailored to the needs of the knowledge users, while balancing timelines and available resources; and,
• Communicate with the knowledge user throughout the review process, or at minimum discuss their expectations for communication in advance.

Rapid reviews are frequently commissioned by a knowledge user to inform a specific decision [11, 25]. Therefore, understanding the reporting requirements of the knowledge user is essential to tailor the rapid review to the knowledge users needed. The knowledge user is likely to be an integral part of the research process, from defining the scope and setting the research question to finalizing the results. As such, they should also be included in the reporting process. Time spent discussing reporting requirements and expectations in advance will help to limit the time required for subsequent revisions and increase utility of the final report. The rapid review report should be tailored to the needs of the knowledge users, while balancing timelines and available resources. Reporting should balance comprehensive accounting of the research process and findings with what is “good enough” or sufficient to meet the requirements of the knowledge users (and/or other stakeholders if important) [12].

#### Special considerations for rapid reviews of health policy and systems research

Health policy and systems research often involves the assessment of complex interventions. Rapid reviews in this field may describe multifaceted or context-specific interventions that may be investigated through a variety of study designs (e.g., controlled before-and-after, interrupted time series, qualitative, or mixed methods studies) [5]. This complexity, and any difficulties encountered during the review process as a result, must be carefully described in the research report, keeping in mind that a wide variety of stakeholders may be interested in the results.

Strategies for reporting rapid evidence products have not been evaluated in comparative studies [5]. As with any knowledge synthesis, reporting for rapid reviews of health policy and systems research should be as comprehensive as possible within the time frame for review completion [12, 14].

#### Reporting checklists and guidelines

Reporting guidelines exist to ensure that research reports contain enough information about the work to make it usable, appraisable, and replicable [28]. Reporting of the rapid review approach and tailoring of the methodology are often inadequate [2, 14, 17]. Details on knowledge user involvement are reported in less than 50% of reports [25]. A detailed assessment of the reporting quality of published rapid reviews, using the Preferred Reporting Items for Systematic Reviews and Meta-Analyses (PRISMA) Statement, also found the reporting to be of poor quality across the included rapid reviews [14]. This assessment found that key decisions in review process and conduct are often presented with insufficient detail or omitted completely. Several publications have noted gaps in the reporting of essential methodological items. For example, many rapid reviews fail to mention the use of a protocol [2], which conflicts with a report that over 90% of organizations producing rapid reviews write and use a protocol [17]. Reporting is often brief or truncated, and methods may be reported in documentation separate from the rapid review report itself [20]. Other items noted in the literature as being poorly reported are the study screening and data collection processes, definitions of study eligibility, methods of assessing risk of bias in or across studies, processes used for syntheses, and limitations in the review process [14].

The literature search located only one tool that provides any reporting guidance specific to rapid reviews: a checklist presented developed by Abrami and colleagues [18]. This checklist reminds authors to provide explanations in key decision areas, and recommends reporting the research question, inclusion criteria, search strategies, inter-rater agreement (if applicable during study identification, calculation of effects, and/or coding of study features), outcome extraction, study features, analysis, interpretation and implications, cautions and limitations, and conclusions. However, the checklist omits several key areas that are worth noting: use of a protocol, inclusion of a structured abstract, explicit identification of the report as a rapid review, internal or external peer review of the review, and critical appraisal of the information included in the review and the types of information sought (e.g., reviews, quantitative or qualitative studies, or other types of research).

To ensure that reporting is complete and transparent, future exploration of reporting (and conduct) guidelines specific to rapid reviews is warranted. Other guidelines and checklists are relevant to rapid reviews, although they focus on the reporting of systematic reviews, such as the PRISMA statement [29, 47]. An extension to PRISMA specific to rapid reviews is currently under development [19]. As there is only a brief published protocol available to-date, it remains unclear if the PRISMA extension for rapid reviews provide guidance specific to health policy and systems reviews. The PRISMA-P extension endeavors to facilitate the reporting of review protocols, which also may be useful to rapid review authors when developing their protocol [29, 30]. Guidance from the Cochrane Rapid Reviews methods Group is specific to the conduct of rapid reviews of interventions and contains no specific reporting or dissemination advice [48]. Other similar organization-specific guidance is available (e.g., manual of the Joanna Briggs Institute [49]); however, this guidance is general and not specific to rapid review. In addition, individual groups or organizations may have internal reporting guidelines or standards. It may be helpful for review authors to check the websites of rapid review producers to see examples of templates and key features [15, 17].

The PRISMA checklist provides a starting point for items to be included in a rapid review report (with certain adjustments specific to the context, such as having the title identify the study as a rapid review, rather than a systematic review). However, it may be more helpful to use the reporting items listed in Table 2, which include some of the PRISMA items but are tailored specifically to rapid reviews. These items may be applicable, depending on the rapid review approach used [4].

**Table 2:** Suggested minimum reporting items for rapid reviews of health policy and systems research

Category	Items to consider
Protocol	• Was a protocol used? • If so, was the protocol made public, published in a journal, and/or registered in advance? (if so, provide reference and/or registration number, or link to protocol)
Overall scope	• Was the scope limited in any way? • Were there a limited number of research or policy questions? • Were the research questions of limited type (e.g., effectiveness only, specific populations)? • Was the number of included studies limited?
Comprehensiveness^a^	• Was the search strategy limited in any way (e.g., number of databases, gray literature, date, setting, language)? • Were there limits on the types of study included (e.g., existing systematic reviews, randomized controlled trials)? • Was textual analysis limited (e.g., no full-text review and/or limits on the number of items extracted)?
Rigor and quality control^a^	• Was the process of dual study selection or dual data extraction modified or omitted? • Was the internal or external review of the final research report limited or omitted?
Synthesis^a^	• Was the assessment of risk of bias or quality of evidence limited or omitted? • Was qualitative or quantitative analysis limited or omitted?
Other^b^	• When making statements about the findings of the rapid review, were the conclusions simplified or omitted? • Is it appropriate to provide a disclaimer^c^ and/or limitations section in context with your findings?

^a^Focus on methodological tailoring

^b^It may help to consider the differences between the present rapid review and the content of a more comprehensive systematic review. This material is likely best provided in the discussion section of the rapid review report, which should include a description of any review limitations

^c^Authors of rapid reviews should consider a disclaimer section in the executive summary, as part of the discussion, or as a note on the cover page, to highlight these limitations and any perceived impact to the review findings. This helps to frame the limitations and to emphasize caution around interpretation [18]

### Dissemination of rapid review findings

Dissemination involves the communication of rapid review findings to specific target audiences, across or within settings, and the tailoring of the knowledge to make it usable to the intended stakeholders [3]. Dissemination may be considered “active” (i.e., efforts using specific strategies and channels) or “passive” (no effort or natural uncontrolled spread) [39]. Little information on passive dissemination specific to rapid review exists. Active approaches are preferred for research dissemination since passive strategies are generally seen as ineffective [39]. Here, we describe typical active dissemination activities undertaken by researchers with a lens on health policy and systems research as there is a paucity of literature related to the specific dissemination of rapid review reports.

Before starting the dissemination process, consider your goal and what the intention of the dissemination process is. A goal can be either for *dissemination only* (i.e., to share your review results with other researchers, funders, policy-makers, or members of the public), or it can be to promote *uptake and implementation* (i.e., to inform or influence decision-making) [3, 9, 40, 50]. Where the goal is *dissemination only*, it is important to identify the targets of the research. These could include researchers, practitioners, policy-makers, patients, and their caregivers or citizens. This will, in turn, inform a targeted active dissemination strategy. A strategy may include presentations at meetings, publications in peer-reviewed publications, or production and sharing of policy briefs, infographics, media releases or social media posts, for example. Preprints (i.e., unvetted versions of research papers) posted to dedicated servers (e.g., medRxiv) provide a platform to enable quick dissemination of rapid review findings [51]. Other fast dissemination mechanisms could also be considered (e.g., Open Science Framework, Zonodo) when more immediate dissemination is indicated [50]. Review authors also need to consider how to engage with policy-makers and other types of decision-makers, such as through knowledge exchange events, to share their research results.

If the goal is to promote *uptake and implementation*, the specific information needs or requests of all knowledge users should guide all dissemination activities. Although the dissemination strategy should focus on meeting the needs of the primary knowledge user, review authors may also consider that if one knowledge user has asked a question, it is likely of interest to others. Review authors may target the primary knowledge user and/or they may also contextualize findings for a broader target audience. Discussion of implementation endeavors and research dissemination frameworks (e.g., Knowledge-to-Action Cycle, the Ottawa Model of Research Use, and the Capability, Opportunity, Motivation, and Behaviour [COM-B] model) is beyond the scope of this literature review, although a number of relevant publications are available for further information [3, 27, 32, 35, 40, 52, 53].

#### Special considerations for rapid reviews of health policy and systems research

Knowledge translation strategies are universally translatable to all forms of research, yet some considerations may be unique to rapid reviews of health policy and systems research. Research into the dissemination of rapid reviews is extremely limited. Two studies of rapid review producers [15, 17] identified variation in research dissemination approaches and tools. In some cases, public dissemination activities may be extremely limited. For example, organizations may choose to post a summary paragraph describing the research, without disseminating a full report [15]. Most rapid review producers (about 70%) chose to disseminate their reports beyond the commissioning individual or body [17]. In deciding the dissemination strategy, influencing factors that have been cited include need for permission from the requester, legal implications or sensitivity of the topic, and type of approach used for the rapid review.

Although traditional methods for research dissemination also apply here, rapid reviews of health policy and systems research may require specific dissemination strategies to reach their target audiences and maximize impact. Some alternative methods to consider are focus groups, public meetings, and open houses. If an advisory board is informing the rapid review process, its members may be able to suggest how to present findings in a way that will reach all potential knowledge users. If it is an expert group, the advisory board may also assist with directly disseminating the results of the rapid review to interested individuals or groups, thereby functioning as knowledge brokers. Barriers faced by LMICs based on conceptual or practical challenges in evidence production and dissemination should also be reflected in comprehensive dissemination strategies [54]. Potential barriers to active dissemination should anticipated, particularly those related to settings where groups or individuals have been marginalized or challenged by historical relationships, mistrust, exploitation, or unequal research partnerships [55]. Additional barriers may be related to the skills or capacity individuals have to appraise or evaluate rapid review findings, or simply time to locate and read evidence reports [17].

There are several simple considerations that may assist with the development of a research dissemination strategy and framing the specific scope to disseminate a rapid review (Table 2). A key factor to consider is the potential impact of the research findings and how generalizable, useful or remarkable they may be to intended knowledge users or other stakeholders [3, 40]. As with any research communication, it is useful to avoid jargon or technical terms and instead, focus on plain language, which does not overstate findings [58].

**Table 3:** Essential questions for developing a rapid review dissemination plan

	**1. Why do you want to raise awareness of your research?:** • to meet the urgent requirement of a knowledge user? • to raise general awareness? • to connect with other researchers? • to generate national or international attention? • to change policy or practice? • to satisfy funders?
	**2. What is interesting about your findings?** • What is novel or different? • Is it a large study? • Are the results contrary to previous evidence? • What is the relevance? • Why now? • Is it a hot topic? • Is it seasonal? • Does your review tap into popular trends? • Are there action-oriented messages that will be relevant to the target audience?
	**3. How might you generate interest in your findings?** • Are you publishing in a journal? • How does the journal generate awareness of papers?
	**4. Who will be interested? Consider the following audiences:** • General public • Patients • Health-care professionals • Researchers • Policy-makers, government • Funders • Corporations
	**5. Should I tailor the message to my audience?** • How can you make your findings interesting to target audiences? • What are your key messages? • Do you need simpler messages for the general public? • How do these differ from messages for policy-makers, researchers?
	**6. What tools can you use to communicate? What can be shared on social media**^**a**^** ?** • Academic publication • Presentations • Meetings and dialogue • Policy briefs • News releases • Preprint publication • Infographics or visual abstracts • Podcasts • Blogs
	**7. Who can best help to deliver your messages?** • Different team members may be good for different platforms (e.g. television interviews, social media, blogging) • Presenters can often be tailored to the audience (e.g. a policy-maker for health system audiences, a researcher for a large research meeting) • A health system stakeholder may be able to talk about your research (e.g. a patient representative, a member of the public or a funding agency spokesperson)
	**8. How will you measure success?** • Number of reads or downloads • Citation metrics^b^ • Altmetrics [36]^c^

^a^Traditional media and social media can be used to publicize research findings to patients and the general public, as well as to researchers, policy-makers, and other audiences [56]. Traditional media include newspapers, radio, television, magazines, and online-only news sites. Social media encompass online and mobile tools, such as Facebook, Twitter, and Instagram, Reddit and more, where users directly create, post, and share content

^b^A variety of metrics can be used to measure the impact of published articles or online content. Citation analysis is used to measure how often a work is cited. One example of a citation metric is the journal impact factor, published in the Web of Science’s Journal Citation Reports, which measures the impact of a journal through its citation by subsequent authors [56]

^c^Altmetrics measure traditional and non-traditional metrics including citations and downloads to web-based scholarly articles, discussions on research blogs, media coverage, citations to public policy documents, and mentions on social networks such as Twitter or Facebook. The more hits from these sources, the higher the Altmetric score [57]

Rapid reviews aim to inform fast-moving policy processes; as such, practical use of the findings by the knowledge users may take priority over academic publication or other broad dissemination approaches. Rapid review producers may also choose to disseminate research findings through publication in peer-reviewed journals, stakeholder meetings or workshops, online summaries and databases, social media posts, video summaries, or e-mail distribution [17] (Table 3). These activities may complement or be in addition to the specific needs of the policy- or decision-makers who requested the review, but their impact on the uptake of information can be limited [34, 59].

#### Knowledge user engagement and dialogue

Clear dialogue and continued engagement are essential to ensure that primary knowledge users’ needs are considered in the rapid review [12, 13, 17, 27]. The practical needs of other knowledge users should also be prioritized when planning for dissemination activities, and discussions should be initiated early. The knowledge users may have a preference for a particular type, or combination of dissemination activities or tools, or require permission for dissemination. In a sample of 29 rapid review programs, factors that appeared to influence the dissemination approach used for rapid evidence product were *turnaround time to complete a report*; *resources available*; *complexity and sensitivity of the research topics*; *and permission from a knowledge user* [17]. Without clear dialogue, important details and opportunities for engagement or dissemination may be lost.

## Discussion

There is a noticeable deficit in guidance to assist evidence producers with reporting and dissemination strategies specific to rapid review, and as such, there is a reliance on tools or approaches that may not be directly applicable or valuable in their entirety for expedited syntheses. There is no guidance available that considers reporting or dissemination of rapid reviews in the area of health systems or policy research. This literature review provides practical recommendations on different approaches and methods for reporting and disseminating expedited knowledge synthesis given the paucity of available research or guidance. As there is no guidance available with a focus on health policy and systems decisions, we hope that we have provided pragmatic considerations and advice that a variety of knowledge users, including researchers, decision-makers, or commissioners and funders of reviews may find helpful. This guidance has been incorporated into the *Rapid Reviews to Strengthen Health Policy and Systems: a Practical Guide* which provides methodological guidance on the conduct of rapid reviews and aims to further their use to inform health policy and systems decisions, with a focus on low-income and middle-income countries (LMICs) [60].

There has been an evolution in the way that rapid evidence products have been produced over the last two decades, including a switch from rapid reviews that evidence producers anticipated would be useful, to approaches driven by, and tailored to specific requests from knowledge users. In the recent literature, colloquial experience from organized rapid review programs show that successful rapid review processes as those that balance the “push-pull tension” of producers and those who commission or request a review, even going so far as to characterize this relationship as a partnership [21]. Approaches have also expanded to include more advanced approaches to statistical synthesis, including rapid network meta-analysis, although the focus has predominantly been on methods development or selection, without additional consideration for reporting or dissemination [22, 23]. Reporting guidance for rapid reviews (PRISMA-RR) will be a useful tool to inform both evidence producers and knowledge users. The strength in this tool will be the rigorous process used in its development, the foundation of existing, best-practice for the reporting of systematic reviews [28], and the adaptation or expansion to consider characteristics and needs specific to rapid reviews [19].

Dissemination must follow basic principles given the scarcity of guidance available specific to rapid review. Dissemination activities and tools should be customized for each review through consideration of the significance of the findings, dissemination goals, target audiences, and anticipated impact or influence of the rapid review. Even with the best plan and intentions for the reporting and dissemination of rapid evidence products, not all decision-makers will use them to inform their decisions [5]. Reports of barriers to uptake of rapid reviews include perceptions that findings are not valid, and limitations related to knowledge of how to identify, access, assess or interpret relevant rapid reviews, and a dearth of skills to assess or interpret rapid reviews [27]. In contrast, enablers are reported to be related to the establishment of partnerships between researchers and policy-makers or health systems managers, and provision of providing education related to the identified barriers [5, 27]. In addition, it can be difficult to gauge how impactful a rapid review contribution was in a decision-making process involving numerous inputs. As the majority of rapid reviews remain unpublished, there is no evidence to quantify the number of rapid reviews that are used for decision-making. Regardless, rapid reviews should aim for clear and transparent reports of methods and findings, as well as consider the aims of dissemination throughout the research synthesis process to ensure that reach, uptake, impact matches expectations of both the producer and user.

The strength in this literature review is that we have presented a broad, practical overview of rapid review reporting and dissemination with a focus on health policy and systems, which updates to our 2017 World Health Organization Guide [60]. Although the review authors are knowledgeable in rapid review literature, our narrow search and non-systematic approach to selecting and screening literature may have missed relevant research publications, which is a limitation. All guidance or suggestions in this article are evidence-based where relevant research was available; however, given the scantiness of available studies, review authors made recommendations that may have been influenced by their personal experiences, preferences, or beliefs.

## Conclusions

There is a paucity of reporting and dissemination guidance for producers of health systems and policy-relevant rapid reviews. Although producers of rapid reviews have access to systematic review reporting and dissemination tools and channels, they may need to prioritize the practical needs of the requesting knowledge user over traditional or academic approaches to reporting and dissemination.

## Supplementary Information

**Additional file 1.** Funding.

## Data Availability

Not applicable.
